# The outbreak of migratory goat’s brucellosis in the Swat ecosystem of Khyber Pakhtunkhwa

**DOI:** 10.4102/ojvr.v90i1.2079

**Published:** 2023-10-25

**Authors:** Nabilla Qayum, Muhammad N. Uddin, Wajid Khan, Habib Un Nabi, Taj-Ud-Din, Muhammad Suleman, Hanif Ur Rahman, Iftikhar Ali, Ahmed Hassan deif, Rafa Almeer, Farman Ullah

**Affiliations:** 1 Center for Biotechnology and Microbiology, University of Swat, Mingora, Pakistan; 2 Department of Livestock, Veterinary Research and Disease Investigation Center (VR&DIC) Balogram, Swat, Pakistan; 3 Department of Livestock, Veterinary Research Institute, Peshawar, Pakistan; 4 Department of Genetics and Development, Columbia University Irving Medical Center, New York, United States of America; 5 School of Life Sciences & Center of Novel Biomaterials, The Chinese University of Hong Kong, Shatin, Hong Kong; 6 Center of Research, Faculty of Engineering, Future University in Egypt, New Cairo, Egypt; 7 Department of Zoology, College of Science, King Saud University, Riyadh, Saudi Arabia

**Keywords:** *Brucella*, serological test, PCR, 16S rRNA, sequencing

## Abstract

Brucellosis is a major threat to public health especially in developing countries including Pakistan. This study reveals the characterisation of *Brucella* species affecting humans and goats in the Swat region of Khyber Pakhtunkhwa, Pakistan. Blood samples were collected from shepherds and goats and analysed by Rose Bengal precipitation test (RBPT), standard plate agglutination test (SPAT), polymerase chain reaction (PCR) and Sanger sequencing of 16S rRNA gene. The findings of the study indicated 24% (36/150) and 11.3% (17/150) positivity for *Brucella abortus* and *Brucella melitensis,* respectively, in human samples. In samples of goats, 26.66% (40/150) were positive for *B. abortus* and 16.66% (25/150) samples were positive *B. melitensis* by SPAT. The species-specific PCR confirmed *B. abortus* in 24% (36/150) of human samples and 26.66% (17/150) of goat samples by targeting the IS711 locus. The remaining seropositive samples were confirmed as *B. melitensis* using IS711 M species-specific primer. The sequences of the amplified fragments of the 16S rRNA gene were blasted, and phylogenetic analysis revealed that *Brucella* species circulating in the Swat district were closely related to *B. melitensis* and *B. abortus* reported from India, China, Philippines, and the United States (US) showing the existence of the possible epidemiological linkage among the *Brucella* species. This study concluded that there was a higher prevalence of *B. abortus* (26.6%) in humans and goats compared to *B. melitensis* (16.6%). These results revealed that the *Brucella* species were circulating in both humans and goats in the study areas. The findings of the study concluded that *B. abortus* and *B. melitensis* were circulating in goats and shepherds with a higher prevalence of *B. abortus* than *B. melitensis*. Furthermore, the *Brucella* species identified in Swat were phylogenetically related to the *Brucella* species reported from India, China, Philippines and the US.

**Contribution:**

The proposed study covers the scope of the journal. The species of the genus *Brucella* affect both animals and shepherds. This study investigates the seroprevalence of brucellosis in shepherds and goats in different geographical areas in the Swat district. The phylogenetic analysis of the *Brucella* spp. identified in Swat showed close relationships to the *Brucella* species reported in India, China, Philippines and the US, which shows the possible epidemiological linkages between the *Brucella* spp.

## Introduction

Pakistan is a South Asian country with an agrarian economy wherein the livestock industry plays a significant role (Kumar, Mittal & Hossain 2008). The country’s livestock population was predicted to be 90.8 million cattle and buffaloes, 109.4m small ruminants (sheep and goats), 1.1m camels and 6.1m equines in 2019. In 2019–2020, this livestock sub-sector provided 60.6% of the value of the agriculture sector and 11.7% of the national gross domestic product (GDP) (Khan et al. 2021). Pakistan is divided into seven administrative regions: Punjab, Sindh, Balochistan, Khyber Pakhtunkhwa (KPK), Gilgit-Baltistan, Azad Jammu and Kashmir (AJK) and the Islamabad Capital Territory (ICT). The bulk of cattle and buffaloes are found in the country’s irrigated parts (Punjab and Sindh), while small ruminants are found mostly in the dry regions. Vaccination and treatment of brucellosis are preferably used for bovines rather than small ruminants (Roth 2011). Khyber Pakhtunkhwa, located in northwest Pakistan, is the third most populous province in terms of both human and livestock populations (Memon et al. 2021). The demography of the area is mostly mountainous, with prominent mountain ranges such as the Hindu Kush and Suleiman mountains. In addition, the population of small ruminants in this area is substantially larger than that of bovines (Khan et al. 2022).

Brucellosis is among the most frequent occupational zoonotic disease in the world, causing tremendous livestock losses and social burdens, particularly in developing countries (Franc et al. 2018). The social burdens of brucellosis include social stigma and discrimination against those infected, as well as economic losses because of decreased productivity of livestock, increased healthcare costs and loss of income. In developing countries, where brucellosis is more prevalent, these social burdens can have a particularly significant impact on the affected individuals’ livelihoods and overall well-being (Cleaveland et al. 2017; McElwain & Thumbi 2017). Because of its slow onset and a lack of symptomatic diagnoses in livestock (Khan & Zahoor 2018), it is generally discovered relatively late, and by that time the entire herd is already affected. Aside from immediate public health effects, the presence of brucellosis may obstruct international trade in animals and animal products (Bagheri Nejad et al. 2020). The disease’s reservoirs include dairy cattle, sheep, goats and pigs, horses, camels and other wild animals are occasionally infected. Recent findings of *Brucella* infection in marine animals (Abalos et al. 2009) and birds (Ali et al. 2020) have provided a new perspective. It is the second most important zoonotic disease in humans after rabies in the world (Abubakar, Mansoor & Arshed 2012). Brucellosis has been eradicated in developed countries, but it remains endemic in some developing countries (Robinson & Production 2003). Worldwide, over half a million cases of the disease are reported annually (Avijgan, Rostamnezhad & Jahanbani-Ardakani 2019). Brucellosis is caused by Gram-negative bacteria of the genus *Brucella* (Mert et al. 2003). Among *Brucella* species, *Brucella abortus* (*B. abortus*) and *Brucella melitensis* (*B. melitensis*) are of great importance because of their high prevalence in both humans and animals (Khamesipour & Momeni 2014). *Brucella melitensis*, in particular, is a reemerging pathogen in the Mediterranean, Arabian Gulf and Middle East regions (Ebid, El Mola & Salib 2020).

Infection in sheep and goats can lead to various health issues such as abortions, weak offspring, reduced milk production, weight loss, infertility and lameness. These problems cause significant economic losses to the global animal industries (Ebid et al. 2020; Franc et al. 2018). Moreover, humans may contract the disease through direct contact with infected animals or consumption of contaminated and unpasteurised dairy products (Mitiku et al. 2021). This can result in acute febrile illness, commonly known as undulant fever. The disease can progress into a chronic form and cause serious complications, affecting the musculoskeletal, cardiovascular and central nervous systems (Galinska & Zagórski 2013). It is well known that individuals who work closely with animals are at a high risk of contracting the disease. Shepherds, abattoir workers, veterinarians, dairy industry professionals and laboratory personnel are all considered to be at risk (Agasthya, Isloor & Prabhudas 2007).

Various control measures have been adopted in different countries based on the elimination of infected animals detected by serological and other diagnostic tests and other control methods based on vaccination (Ebid et al. 2020). Presently, three categories of diagnostic tools are used for the detection of *Brucella* species: these are conventional, serological and molecular-based diagnostic tools (Mabe et al. 2022). The conventional tools are laborious and time-consuming and also pose a high risk of causing infection to the laboratory personnel as *Brucella* is contagious, and its handling requires biosafety level III or IV laboratories for its isolation and culture. The common serological techniques include the Rose Bengal precipitation test (RBPT), standard plate agglutination test (SPAT), Coombs test, immune capture test and enzyme-linked immunosorbent assay (ELISA). The World Organization for Animal Health (OIE) recommends the use of two serological tests for the diagnosis of brucellosis in animals intended for international trade: the Rose Bengal test (RBT) and the indirect enzyme-linked immunosorbent assay (iELISA) (McGiven et al. 2006). Currently, these conventional techniques are replaced by a molecular technique, polymerase chain reaction (PCR), used for the detection of *Brucella* and its species because of its high sensitivity and specificity (Marín et al. 2007). Polymerase chain reaction has proven to be the most sensitive diagnostic tool for both *B. abortus* and *B. melitensis* in human patients (Kamal et al. 2013). Brucellosis is distributed worldwide and is more common in countries with poor animal and public health hygiene programmes. Although it has been eradicated from many developed countries, it remains an endemic disease in some regions including Pakistan. In addition, the lack of studies on the prevalence of brucellosis in shepherds and migratory goats in the Swat region of Pakistan needs to be updated on the infection rate. The objective of this study was to establish the seroprevalence of brucellosis in shepherds and goats in Swat. Furthermore, the causative agents of brucellosis were characterised based on the Sanger sequencing of the 16S rRNA gene.

## Research methods and design

### Study area

**FIGURE 1:**
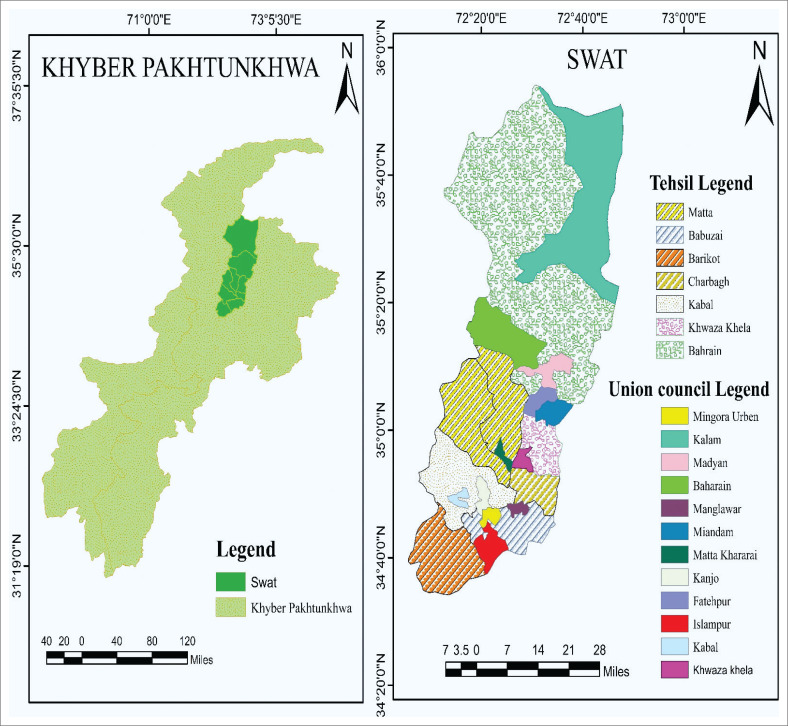
Demography of the Swat district.

This study was performed on migratory goats and shepherds in different tehsils of the Swat district. It is a cultural tradition for shepherds of this area to migrate with their flocks during the winter months and this practice has been passed down through generations. Previous data about only household domestic small ruminants are available, and there is little knowledge about the seasonal migratory goats in the area. Detailed information regarding brucellosis was collected on a pre-designed questionnaire for both migratory shepherds and goats. Samples were gathered from goats and shepherds who had returned to the area after seasonal migration from seven tehsils, namely Babuzai, Bahrain, Barikot, Charbagh, Kabal, Khwazakhela and Matta, in the Swat district. The demography of the under study area is shown in Figure 1.

### Sample collection

Samples were collected from the goat flocks, as well as from the shepherds and their family members who returned to the area after seasonal migration along with their goat flocks. Blood samples (*n* = 300) were collected from shepherds and goats (shepherds, n = 150 and goats, n = 150) by following standard procedures without any animal harm (Wang et al. 2015). A total of 3 mL to 5 mL of blood samples were collected aseptically from the jugular vein of goats and cephalic vein of shepherds. To maintain the integrity of red blood cells, disposable syringes were utilised to collect blood samples, which were then introduced into both Gel tubes and EDTA tubes without applying any pressure. Proper labeling was employed, and the blood samples were transported to the laboratory of the Centre for Biotechnology and Microbiology at the University of Swat for further analysis. The blood samples were refrigerated overnight until they were processed.

### Sample processing

Blood samples were kept in an upright position for 30 min in a cool place and then centrifuged at 3000 revolutions per minute (rpm) for 20 min. The serum was collected by a micropipette and placed in Eppendorf tubes. Serum samples and blood samples were kept at −20 °C until used, as previously processed by Al-Garadi et al. (2011). Blood samples were analysed by RBPT, SPAT and PCR.

### Serological tests

#### Rose Bengal Plate Test

The initial screening process involved using the coloured Rose Bengal Plate Test (RBPT) antigens following the protocol outlined by OIE in 2014. To ensure a uniform antigen suspension, both the serum and antigen were equilibrated to room temperature and vigorously agitated. A single drop of 30 μL (0.03 mL) of serum sample and 40 μL (0.04 mL) of Rose Bengal antigen were added to the same slide plate, creating a zone of approximately 3 cm in diameter. Using separate micropipettes, the samples were mixed thoroughly with a mixing stick. The slide plates were then rotated gently in a clockwise and counterclockwise motion for 4 min. Afterwards, the slide plates were examined under a bright light to detect any agglutination, which would appear as small clumps or dots. The results were interpreted based on the specific RBPT kit instructions and established diagnostic criteria for goats, as reported by Ebid et al. (2020).

#### Standard Plate Agglutination Test

The SPAT was performed following the standardised protocols of OIE in 2014., Briefly, both the antigen and serum were equilibrated to room temperature. An 80 μL sample of each serum was then placed on a glass slide. Next, 30 μL of antigen was added to create a homogeneous mixture. Using a mixing stick, the serum and antigen were mixed thoroughly, forming a circular zone of approximately 3 cm in diameter. The slide was gently rotated in a circular motion, both clockwise and counterclockwise, for 3 min to 4 min. Positive samples exhibited agglutination, while negative samples displayed no agglutination, as reported earlier by Ebid et al. (2020).

### Molecular identification

All samples that tested positive for RBPT and SPAT were further confirmed by PCR. *Brucella* deoxyribonucleic acid (DNA) was amplified and detected by PCR using the protocol described by Romero et al. (1995). Gel electrophoresis for PCR product was performed by following the standardised methodology of Ahmad and Rahman (2019). Briefly, serum samples were used to obtain genomic DNA using the QIAamp DNA Mini Kit (Qiagen kit, Germany) following manufacturer protocol. The DNA was extracted and purified from the pellet via DNA Purification Kit (GeneJET Genomic; Thermofisher Scientific-K0721). After the DNA purification, a PCR assay was performed for targeting the genus *Brucella* using the genus-specific primer (BCSP31 gene (BCSP31-PCR). The positive samples were then subjected to PCR (Thermo Fisher Scientific) using species-specific primers (IS711AB locus [IS711-PCR] for *B. abortus* and IS71M locus for *B. melitensis*). The thermocycler programme was adjusted for 40 cycles as; DNA denaturation for 1 min at 94 °C – annealing of primer for 30 s at 60 °C – Elongation for 1 min at 72 °C – Final elongation step of 10 min at 72 °C. At the end of the thermocycler programme, the amplified product was run on 2% agarose gel in gel electrophoresis for 60 min at 110 volt.

The positive samples for brucellosis were subjected to 16S rRNA PCR and sequencing. The universal primers of 16S rRNA (Forward 5’-GTG-CCA-GCA-GCC-GCCGTA-ATA-C-3′) and reverse (5’-TGG-TGT-GAC-GGG-CGG-TGT-GTA-CAA-3′) were used. The PCR protocol was optimised for 25 μL final reaction volume, containing 12.5 μL of master mix, DNA template (5 μL), PCR-grade water (5.5 μL), and each primer of 1 μL. The reaction was run for 40 cycles under the optimised condition of PCR (initial denaturation and final denaturation at 95 °C for 5 min and 30 s, respectively, annealing temperature at 52 °C for the 30 s, initial extension and final extension at 52 °C for 30 s and 5 min, respectively). The PCR products were visualised through gel electrophoresis by following the methodology of Ntirandekura et al. (2020) with some modifications. Before sequencing, the PCR products were first purified through a purification kit (GFXTM PCR DNA and Gel band purification) following the manufacturer’s protocol. The purified samples were then subjected to Sanger sequencing. The sequencing was performed using Cycle Sequencing Kit (BigDye^®^ Terminator version 3.1). The final sequencing reaction volume was 10 μL, containing 2 μL of sequencing buffer (5X) and sequencing primers (forward and reverse primers of 16s rRNA gene). Sequencing was performed twice for each primer with the optimised sequencing condition (96 °C for 1 min, followed by 25 cycles of denaturation at 96 °C for 10 s, 50 °C for 5 s and 60 °C for 4 min). Next, ethanol precipitation was used for cleaning the sequencing products (Ntirandekura et al. 2020). After precipitation, the tubes containing the sequencing products were centrifuged for 30 min at 13 000 rpm. The pellets were washed with ethanol (70%) after discarding the supernatant and then centrifuged at 13 000 rpm for 30 min. The samples were then treated with Hi-Di^™^ formamide and then loaded on a DNA analyser (ABI 3730). The analysis was performed according to the manufacturer’s protocol.

### Sequence analysis

The Bioedit software was used to visualise the sequences obtained from Sanger sequencing of BSCP31 isolates from human and goat samples. Chromatogram quality was assessed, low-quality bases were trimmed, and all ambiguities were corrected. Consensus sequences were generated by aligning the forward and reverse sequences of each sample. NCBI BLAST tool was used to identify similar sequences, and sequences with 80% or greater similarity were downloaded from Gene bank. The maximum likelihood method and the Jukes-Cantor model were employed to infer the phylogenetic relationship. Consensus bootstrap with 100 replicates was used to infer the evolutionary history of the eight nucleotide sequences analysed. The final dataset contained 1002 positions for the goat sample and 986 positions for the human sample. Evolutionary analyses were conducted using MEGA X (Girault et al. 2022).

### Statistical analysis

The raw data collected from pre-designed questionnaire for both migratory shepherds and goats was manually entered in a Microsoft Excel spreadsheet (2016 Version). The data were analysed using the Statistical Package for Social Sciences (SPSS) software version 23 (IBM SPSS version 23). Chi-square test was performed to check the statistical significance between categorical variables and prevalence of brucellosis. The results were considered statistically significant if *p* ≤ 0.05 at 95% confidence interval.

### Ethical considerations

The Ethical Research Committee (ERC) of the University of Swat approved the research project entitled ‘The episode of human and migratory goats Brucellosis in the Swat Ecosystem of Khyber Pakhtunkhwa, Pakistan’ (No. UoS/ORIC/2021/13).

No animal was harmed during the sample collection process and the data of shepherds was kept confidential.

## Results

The data presented in Table 1 depicts the seroprevalence of brucellosis in different areas of Swat using RBPT and SPAT. The RBPT results showed a higher prevalence of brucellosis in Tehsil Kabal (10/20, 50%), followed by Tehsil Matta and Charbagh with prevalence rates of 40% (8/20). The lowest prevalence was found in Tehsil Babozai (8/20, 26.7%). The overall prevalence of brucellosis in Swat was 35.3% (53/150). The SPAT results showed the highest prevalence of *Brucella abortus* in Kabal (7/20, 35%), followed by Matta (6/20, 40%), and the lowest prevalence in Babozai (5/30, 16.7%). *Brucella melitensis* was detected in 3/20 samples from Kabal and Charbagh, with a prevalence rate of 15%, and in 10% of samples from other regions. The overall prevalence of *B. abortus* and *B. melitensis* was 24% (36/150) and 11.3% (17/150), respectively. There was no significant difference (*p* ≥ 0.05) in area-wise seroprevalence of brucellosis in Swat among migratory shepherds.

**TABLE 1:** Area-wise seroprevalence of brucellosis in shepherds.

Tehsil name	RBPT			SPAT					
	No. of positive samples		% positive samples	*B. abortus*			*B. melitensis*		
				No. of positive samples		% positive samples	No. of positive samples		% positive samples
	*n*	*N*		*n*	*N*		*n*	*N*	
Babozai	8	30	26.7	5	30	16.7	3	30	10.0
Bahrain	7	20	35.0	5	20	25.0	2	20	10.0
Barikot	6	20	30.0	4	20	20.0	2	20	10.0
Charbagh	8	20	40.0	5	20	25.0	3	20	15.0
Kabal	10	20	50.0	7	20	35.0	3	20	15.0
Khwazakhela	6	20	30.0	4	20	20.0	2	20	10.0
Matta	8	20	40.0	6	20	30.0	2	20	10.0

**Total**	**53**	**150**	**35.3**	**36**	**150**	**24.00**	**17**	**150**	**11.33**

Note: Chi-square test: RBPT *p* ≥ 0.05 = 3.7, SPAT *p* ≥ 0.05 = 4.2. There is no significant difference in area-wise seroprevalence of brucellosis as *p* ≥ 0.05 for both RBPT and SPAT in area-wise seroprevalence of brucellosis in shepherds.

RBPT, Rose Bengal precipitation test; SPAT, standard plate agglutination test; No., number.

The seroprevalence of brucellosis among shepherds’ samples was analysed by age group, and the results are presented in Table 2. The highest prevalence rate of 41.3% (26/63) was found in the age group of 21–30 years, followed by the age group of 31–40 years with a prevalence rate of 33.3% (11/33). The lowest prevalence rate of 29.6% (16/54) was observed in the age group of 11–20 years. Table 2 also displays age-wise seroprevalence of *B. abortus* and *B. melitensis* detected through SPAT. The highest prevalence of *B. abortus* was found in the age group of 21–30 years with a prevalence rate of 31.8% (20/63), followed by the age group of 11–20 years with a prevalence rate of 18.5% (10/54). The lowest prevalence rate of 18.2% (6/33) for *B. abortus* was observed in the age group of 31–40 years. The prevalence rate of *B. melitensis* was highest in the age group of 31–40 years with a prevalence rate of 15.1% (5/33), followed by the age group of 11–20 years with a prevalence rate of 11.1% (6/54), and the age group of 21–30 years with a prevalence rate of 9.5% (6/63). There was no significant difference (*p* ≥ 0.05) found in age-wise seroprevalence of brucellosis for shepherds.

**TABLE 2:** Age-wise seroprevalence of brucellosis in shepherds.

Age group in years	RBPT			SPAT					
	No. of positive samples		% positive samples	*B. abortus*			*B. melitensis*		
				No. of positive samples		% positive samples	No. of positive samples		% Positive samples
	*n*	*N*		*n*	*N*		*n*	*N*	
11–20	16	54	29.6	10	54	18.5	6	54	11.1
21–30	26	63	41.3	20	63	31.8	6	63	9.5
31–40	11	33	33.3	6	33	18.2	5	33	15.1

**Total**	**53**	**150**	**35.33**	**36**	**150**	**24.00**	**17**	**150**	**11.33**

Note: Chi-square test: RBPT *p* ≥ 0.05 = 2.6, SPAT *p* ≥ 0.05 = 4.6. There is no significant difference in age-wise seroprevalence of brucellosis as *p* ≥ 0.05 for both RBPT and SPAT in age-wise seroprevalence of brucellosis in shepherds.

RBPT, Rose Bengal precipitation test; SPAT, standard plate agglutination test; No., number.

The results presented in Table 3 show the gender-wise seroprevalence of brucellosis in human samples tested using RBPT. Females had a higher prevalence rate (42%, 37/88) compared with males (25.8%, 16/62). Table 3 also displays the gender-wise seroprevalence of *B. abortus* and *B. melitensis* tested using SPAT. The prevalence of *B. abortus* was higher in females (31.8%, 28/88) than in males (12.9%, 8/62), whereas the prevalence of *B. melitensis* was higher in males (12.9%, 8/62) than in females (10.2%, 9/88). No significant difference (*p* ≥ 0.05) was found in the gender-wise seroprevalence data for shepherds.

**TABLE 3:** Gender-wise seroprevalence of brucellosis in shepherds.

Gender	RBPT			SPAT					
	No of positive samples		% positive samples	*B. abortus*			*B. melitensis*		
				No of positive samples		% positive samples	No of positive samples		% positive samples
	*n*	*N*		*n*	*N*		*n*	*N*	
Male	16	62	25.8	8	62	12.9	8	62	12.9
Female	37	88	42.0	28	88	31.8	9	88	10.2

**Total**	**53**	**150**	**35.33**	**36**	**150**	**24.00**	**17**	**150**	**11.33**

Note: Chi-square test: RBPT *p* ≥ 0.05 = 0.4, SPAT *p* ≥ 0.05 = 4.1. *p* ≥ 0.05 for both RBPT and SPAT in gender-wise seroprevalence of brucellosis in shepherds.

RBPT, Rose Bengal precipitation test; SPAT, standard plate agglutination test; No., number; *B. abortus, Brucella abortus; B. melitensis, Brucella melitensis*.

The seroprevalence of brucellosis in goat samples across different areas was displayed in Table 4. The highest prevalence rate of 60% (12/20) was observed in Tehsil Bahrain, followed by Tehsil Matta and Barikot with 50% (10/20) prevalence each. Tehsil Kabal and Khwazakhela also had a considerable prevalence rate of 45% (9/20). The lowest prevalence rate of 23.3% (7/30) was recorded in Tehsil Babozai. The data in Table 4 also presented the area-wise seroprevalence of *B. abortus* and *B. melitensis* on SPAT. The highest prevalence rate of *B. abortus* was observed in Tehsil Bahrain and Tehsil Barikot with 35% (7/20) prevalence each, followed by Tehsil Kabal and Tehsil Khwazakhela with 30% (6/20) prevalence each. The lowest prevalence rate of *B. abortus* was found in Tehsil Babozai with 13.3% (4/30) prevalence. The prevalence rate of *B. melitensis* was highest in Tehsil Matta and Bahrain with 25% (5/20) prevalence each, while the lowest prevalence rate of *B. melitensis* was observed in Tehsil Babozai with 10% (3/30) prevalence. The chi-square test value was greater than *p* ≤ 0.05, indicating no significant difference in the area-wise seroprevalence of brucellosis in goats.

**TABLE 4:** Area-wise seroprevalence of brucellosis in goats.

Tehsil name	RBPT			SPAT					
	No. of positive samples		% positive samples	*B. abortus*			*B. melitensis*		
				No. of positive samples		% positive samples	No. of positive samples		% positive samples
	*n*	*N*		*n*	*N*		*n*	*N*	
Babozai	7	30	23.3	4	30	13.3	3	30	10.0
Bahrain	12	20	60.0	7	20	35.0	5	20	25.0
Barikot	10	20	50.0	7	20	35.0	3	20	15.0
Charbagh	8	20	40.0	5	20	25.0	3	20	15.0
Kabal	9	20	45.0	6	20	30.0	3	20	15.0
Khwazakhela	9	20	45.0	6	20	30.0	3	20	15.0
Matta	10	20	50.0	5	20	25.0	5	20	25.0

**Total**	**65**	**150**	**43.33**	**40**	**150**	**26.66**	**25**	**150**	**16.66**

Note: Chi-square test: RBPT *p* = 0.2, SPAT *p* = 0.6. *p* ≥ 0.05 for both RBPT and SPAT in area-wise seroprevalence of brucellosis in goats.

RBPT, Rose Bengal precipitation test; SPAT, standard plate agglutination test; No., number; *B. abortus, Brucella abortus; B. melitensis, Brucella melitensis*.

Table 5 presents data on gender-wise seroprevalence of brucellosis in goat samples using RBPT and SPAT. The prevalence rate was found to be higher in female goats (55/107, 51.4%) than in male goats (10/43, 23.2%). The table also shows the gender-wise seroprevalence of *B. abortus* and *B. melitensis* in goats using SPAT. Out of 150 samples, 40 (26.6%) were positive for *B. abortus* and 25 (16.65%) were positive for *B. melitensis*. Female goats had a higher rate of *B. abortus* prevalence (39/107, 36.5%) compared with males (1/43, 2.3%), while *B. melitensis* prevalence was higher in males (9/43, 20.9%) than in female goats (16/107, 14.9%). There was no significant difference (*p* ≥ 0.05) found in the gender-wise seroprevalence data for brucellosis in goats.

**TABLE 5:** Gender-wise seroprevalence of brucellosis in goats.

Gender	RBPT			SPAT					
	No. of positive samples		% positive samples	*B. abortus*			*B. melitensis*		
				No. of positive samples		% positive samples	No. of positive samples		% positive samples
	*n*	*N*		*n*	*N*		*n*	*N*	
Male	10	43	23.2	1	43	2.3	9	43	20.9
Female	55	107	51.4	39	107	36.5	16	107	14.9

**Total**	**65**	**150**	**43.33**	**40**	**150**	**26.66**	**25**	**150**	**16.66**

Note: Chi-square test: RBPT *p* = 0.3, SPAT *p* = 0.6. *p* ≥ 0.05 for both RBPT and SPAT in gender-wise seroprevalence of brucellosis in goats.

RBPT, Rose Bengal precipitation test; SPAT, standard plate agglutination test; No., number; *B. abortus, Brucella abortus; B. melitensis, Brucella melitensis.*

**FIGURE 2:**
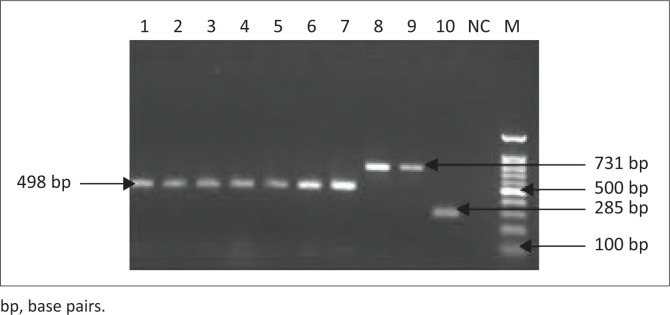
Polymerase chain reaction amplified fragments of gene BSCP31 and IS711 locus in *Brucella* genus, *Brucella abortus* and *Brucella melitensis* with an amplicon size of 285 bp, 498 bp and 731 bp, respectively. Lane: 1 to 10 positive serum, lane NC: negative control, lane M: DNA ladder.

**FIGURE 3:**
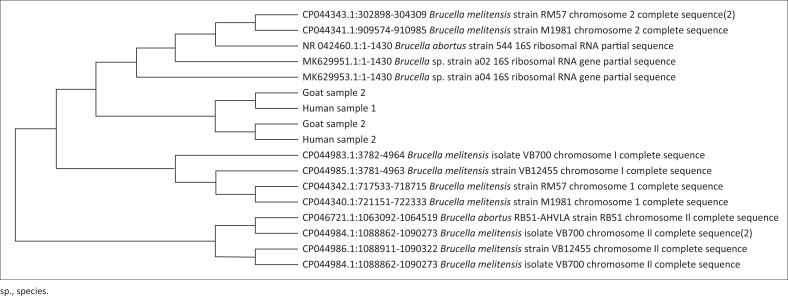
Phylogenetic analysis of Brucella spp. from Swat region in relation with other reported species of Brucella based on 16S rRNA gene sequences by maximum likelihood method.

After performing the initial screening of samples on SPAT, sets of primers were used for the molecular identification and characterisation of *Brucella* species using PCR assay. The species-specific primer (IS711 AB) targeted the IS711 locus in the genome and produced an amplicon size of 498 base pairs (bp) and thus confirmed the presence of *B. abortus* in 24% of human samples and 26.66% of goat samples. The remaining seropositive samples (11.3% human and 16.66% goats) were confirmed for the presence of *B. melitensis* with an amplicon size of 731 bp (Figure 2). The seropositive samples of brucellosis were then subjected to PCR-based amplification of 16S rRNA genes and Sanger sequencing to characterise the *Brucella* species circulating in the ecosystem of Swat. The amplified fragments of 16S rRNA genes were then sequenced by Sanger sequencing and the sequences obtained were blasted for similarity search. The different parameters of query sequences (E value, percent identity, etc.) revealed homology with the sequences of different species of *Brucella* (*B. melitensis, B. abortus*) deposited in the Gene bank. After cleaning the sequences, phylogenetic analysis was performed to characterise the *Brucella* species from the Swat region. The phylogenetic tree of the 16S rRNA gene sequences showed that *Brucella* spp. from the Swat region were grouped into two clades and two branches, all closer to *B. melitensis* and *B. abortus* reported from the different areas of the world (Figure 3).

## Discussion

The management and diagnosis of brucellosis are challenging because of prolonged culturing time and contagious nature of *Brucella* species (Shenoy, Jaiswal & Vinod 2016). Therefore, serological and molecular techniques such as RBPT, SPAT and PCR are promising alternatives for the timely diagnosis of fastidious microorganisms such as *Brucella* species. Serological tests such as RBPT and SPAT were used by previous researchers to find out the seroprevalence of brucellosis in animals as well as in humans (Khan et al. 2022; Niaz et al. 2021). This study also documented the seroprevalence of brucellosis in shepherds and goats based on RBPT and SPAT; the results were further confirmed by species-specific PCR.

This study documented 35.3% seropositivity of human brucellosis in the study area, with 24% and 11.3% positivity for *B. abortus* and *B. melitensis*, respectively (Table 1). The findings of the present study are in line with the results of Thapa et al. (2018). In contrast, the results showed a high prevalence of brucellosis than the study conducted in Malakand district of Khyber Pakhtunkhwa by Niaz et al. (2021) and Alkahtani et al. (2020). Their results showed 18.8% and 5.3% positivity for brucellosis, respectively. Niaz et al. (2021) reported the majority of the positive cases from rural areas. The difference in results may be because of sampling technique as they use samples from people living in rural areas involved in animal husbandry, and this study involved samples from shepherds only. The results of previous researchers provide evidence that *B. abortus* is more prevalent in humans than *B. melitensis* in various regions around the world (Corbel 1989; Musallam et al. 2016).

The highest prevalence rate of 41.3% was found in the age group of 21–30 years in humans (Table 2). Niaz et al. (2021) also found a high prevalence rate of brucellosis in the age group 21–30 years. The overall prevalence of brucellosis in adult population was 35.3%, which is much higher than the study conducted by Mantur et al. (2004), they documented 495 cases of brucellosis in adult population with a prevalence rate of 1.8% using blood samples. The difference may be because of difference in sampling populations as this study collected samples only from shepherds and they collected samples from random people. Females had a higher prevalence rate compared with males. Our results are in contradiction to the study performed by Al-Tawfiq and AbuKhamsin (2009), they reported three times more prevalence of brucellosis in males than females in Kuwait and Eastern Saudi Arabia. The possibilities for this difference may be the difference in sampling population and differences in culture of females’ endorsement in grazing animals.

The seroprevalence in goats showed 43.3% prevalence rate with high prevalence of *B. abortus* as compared with *B. melitensis* (Table 4). Our results of 43.33% prevalence of brucellosis in 150 goat samples are in line with the findings of Thapa and Maharjan (2018). These results are in contrast with the previous data (Miller et al. 2016). Miller et al. (2016) reported overall prevalence of brucellosis was 17.5% in goats. The observed difference in the data may be attributed to various factors, including the different livestock management systems, grazing techniques and migratory patterns as well as the awareness of the shepherds about brucellosis in the area. Moreover, the sampling method employed was a convenience sampling method where samples were collected randomly, making it difficult to estimate the significance of the observed differences in the data.

Serological tests are known for their specificity and sensitivity, but molecular techniques such as PCR are becoming increasingly popular because of their rapid and accurate detection of brucellosis. Polymerase chain reaction is often considered the best and most reliable method for diagnosing and confirming brucellosis in both animals and humans, as previously reported (Mol et al. 2020). In this study, we used species-specific primer targeting the IS711 locus in the genome to confirm the presence of *B. abortus* and *B. melitensis* in human samples and goat samples. Other researchers have also used the similar set of primers and obtained the similar size of amplified fragment from the genome of *Brucella* species in PCR assay; thus this study supports the previous finding (Ali et al. 2014). Furthermore, the accuracy and reliability of the species specific primers for detecting the *Brucella* species in blood sera were also reported by Ahmad and Rahman (2019).

In this study, we are also reporting the PCR-based amplification of 16S rRNA genes and Sanger sequencing for the characterisation of *Brucella* spp. in goats and shepherds in the Swat region of Khyber Pakhtunkhwa. Positive samples of brucellosis were subjected to Sanger sequencing and the sequences obtained were blasted for similarity search. The E value, percent identity and the query cover of each sequence revealed its similarity with the different species of *Brucella* (*B. melitensis, B. abortus*) deposited in the Gene bank, which showed that they shared homology from common ancestry and similar structure (Ntirandekura et al. 2020). The phylogenetic tree of the 16S rRNA gene sequences showed that *Brucella* spp. from the Swat region were grouped into two clades and two branches, all closer to *B. melitensis* and *B. abortus* reported from India, China, Philippines and the US. However, they also showed divergence from other species isolated in India, China and the US. The previous studies reported the identification and characterisation of *Brucella* in the region by targeting other genes for sequencing (Hoffman et al. 2016; Mathew et al. 2017; Mugizi et al. 2015). As per the authors knowledge, this is the first research study that reports the identification and characterisation of *Brucella* species in the Swat region of Khyber Pakhtunkhwa, Pakistan.

Furthermore, the grouping of *Brucella* spp. into two clades and two branches reported in Swat, representing the existence of genetic heterogeneity among the species in the targeted area. On the other hand, the phylogenetic tree further revealed that *Brucella* species circulating in district Swat were closer to *B. melitensis* and *B. abortus* reported from India, China, Philippines and the US showing the existence of the possible epidemiological linkage among the *Brucella* species.

## Conclusion

This study concluded that *Brucella* species were circulating in livestock and shepherds in this region. *Brucella abortus* was the most prevalent species in both shepherds and animals in the study area. Females of goats as well as humans were more vulnerable to brucellosis. Rose Bengal precipitation test and SPAT can be used for the rapid detection and early diagnosis of brucellosis as their sensitivity and specificity were confirmed by PCR. Furthermore, the *Brucella* spp. identified in Swat were phylogenetically related to the *Brucella* spp. reported from India, China, Philippines and the US, thus showed possible epidemiological linkages between *Brucella* spp.
