# Corrigendum: The outbreak of seasonal goat’s brucellosis in the Swat ecosystem of Khyber Pakhtunkhwa

**DOI:** 10.4102/ojvr.v91i1.2150

**Published:** 2024-02-29

**Authors:** Nabilla Qayum, Muhammad N. Uddin, Wajid Khan, Habib Un Nabi, Taj-Ud-Din, Muhammad Suleman, Hanif Ur Rahman, Iftikhar Ali, Ahmed M. Hassan, Rafa Almeer, Farman Ullah

**Affiliations:** 1 Center for Biotechnology and Microbiology, University of Swat, Mingora, Pakistan; 2 Department of Livestock, Veterinary Research and Disease Investigation Center (VR&DIC) Balogram, Swat, Pakistan; 3 Department of Livestock, Veterinary Research Institute, Peshawar, Pakistan; 4 Department of Genetics and Development, Columbia University Irving Medical Center, New York, United States of America; 5 School of Life Sciences & Center of Novel Biomaterials, The Chinese University of Hong Kong, Shatin, Hong Kong; 6 Center of Research, Faculty of Engineering, Future University in Egypt, New Cairo, Egypt; 7 Department of Zoology, College of Science, King Saud University, Riyadh, Saudi Arabia

In the published article, Qayum, N., Uddin, M.N., Khan, W., Un Nabi, H., Taj-Ud-Din., Suleman, M. et al. 2023, ‘The outbreak of migratory goat’s brucellosis in the Swat ecosystem of Khyber Pakhtunkhwa’, *Onderstepoort Journal of Veterinary Research* 90(1), a2079. https://doi.org/10.4102/ojvr.v90i1.2079, there was an error with the spelling of two of the authors’ names.

Instead of:

Ahmed Hasssan deif and Taj Ud Din

It should be:

Ahmed M. Hassen and Taj-Ud-Din

The authors apologise for this error. The correction does not change the study’s findings of significance or overall interpretation of the study’s results or the scientific conclusions of the article in any way.

